# Comparing the ORBIT and HAS-BLED bleeding risk scores in anticoagulated atrial fibrillation patients: a systematic review and meta-analysis

**DOI:** 10.18632/oncotarget.19858

**Published:** 2017-08-03

**Authors:** Cen Wang, Ye Yu, Wengen Zhu, Jianhua Yu, Gregory Y.H. Lip, Kui Hong

**Affiliations:** ^1^ Department of Cardiovascular Medicine, the Second Affiliated Hospital of Nanchang University, Jiangxi, China; ^2^ The Jiangxi Key Laboratory of Molecular Medicine, Jiangxi, China; ^3^ University of Birmingham Centre for Cardiovascular Sciences, City Hospital, Birmingham, United Kingdom

**Keywords:** atrial fibrillation, ORBIT, HAS-BLED, anticoagulation, major bleeding

## Abstract

**Background:**

The HAS-BLED and ORBIT scores have been proposed to assess bleeding risk in anticoagulated atrial fibrillation patients. We performed a systematic review and meta-analysis to compare the predictive ability by using these two scores.

**Materials and Methods:**

We searched the Cochrane Library, Elsevier and PubMed databases for related studies. Statistical analysis was performed with Revman 5.3 Manager software. We chose the C-statistic to reflect the diagnostic value.

**Results:**

In our seven selected studies, the pooled C- statistic of continuous variables for major bleeding was 0.65 (0.60,0.69) for ORBIT and 0.63 (0.60,0.66) for HAS-BLED. Compared with HAS-BLED, more anticoagulated AF patients (88.45% versus 32.59%) and major bleeding events (75.57% versus 25.57%) were categorized as low risk. The ORBIT score had a 1.21, 1.73 and 1.44-fold elevated risk of major bleeding in the low, intermediate and high risk strata respectively. Calibration analysis demonstrated that the ORBIT score under-predicted major bleeding in the low, intermediate, and high risk stratifications, where a odds ratio of 0.64 (0.37–1.10), 0.63 (0.38–1.05) and 0.64 (0.38–1.06), respectively.

**Conclusions:**

Compared with HAS-BLED , the ORBIT score does not perform better in predicting major bleeding events in anticoagulated atrial fibrillation patients. More anticoagulated AF patients and major bleeding events were categorized as low risk when using ORBIT.

## INTRODUCTION

Oral anticoagulants (OAC) are the cornerstones of atrial fibrillation (AF) management for preventing of stroke, thromboembolic events and mortality [1–3]. Bleeding is the major downside of OAC therapy [4, 5].

Many common clinical features are associated with an increase in bleeding risk, and the more common features (many of which are potentially reversible or correctable) have been used to formulate various bleeding risk stratification schema [5]. Nonetheless bleeding risk scores have been subject to misuse, as the objective of performing a bleeding risk assessment is to ‘flag up’ patients potentially at risk of bleeding for more careful review and follow up, as well as to address the potentially reversible bleeding risk factors [6].

Of the various published bleeding risk scores, the HAS-BLED score (hypertension, abnormal renal/liver function, stroke, bleeding history or predisposition, labile international normalized ratio [INR], elderly [age ≥ 65 years], and drugs/alcohol concomitantly) has received widespread attention since it was first proposed in 2010 [7]. In 2015, O’Brien et al. derived and validated a simple score, ORBIT [8] (older [age ≥ 74 years], reduced hemoglobin/hematocrit/history of anemia, bleeding history, insufficient kidney function, and treatment with antiplatelet), which could be adapted to both VKA and non-VKA users. The ORBIT score was derived from the ORBIT-AF registry [9], with 10,098 AF patients taking warfarin and dabigatran, and validated in the ROCKET-AF trial population (treated with warfarin or rivaroxaban) [8]. According to the ORBIT score, AF patients were categorized as ‘low’, ‘intermediate’, and ‘high’ risk, based on score points ‘0-2’, ‘3’, and ‘≥ 4’, respectively. Table 1 summarises the components of the HAS-BLED and ORBIT scores.

**Table 1:** The risk predictors and risk stratifications of bleeding scores

	Risk predictors	Scoring system	Risk stratification
**ORBIT**	Older age (≥ 74 years)		
	Reduced hemoglobin/anemia		Low risk 0–2
	Bleeding history	1 point for each risk factor	Intermediate risk 3
	Insufficient kidney function		High risk ≥ 4
	Treatment with antiplatelet		
**HAS-BLED**	Hypertension		
	Abnormal renal and/or liver function		
	Stroke		Low risk 0–1
	Bleeding history	1 point for each risk factor	Intermediate risk 2
	Labile INR		High risk ≥ 3
	Elderly (≥ 65 years)		
	Drugs or alcohol concomitant		

It is unclear whether the ORBIT or HAS-BLED score has a better predictive ability for bleeding in AF patients [8, 10–15]. Our previous meta-analysis compared the HAS-BLED score with several bleeding risk scores (not include the ORBIT score) and showed that the HAS-BLED score had a better ability to predict bleeding risk than the other selected bleeding risk scores [16].

Given the recent interest in the ORBIT score, we performed a systematic review and meta-analysis to compare the predictive ability of bleeding risks using the ORBIT and HAS-BLED scores.

## MATERIALS AND METHODS

### Inclusion and exclusion criteria

We selected studies according to the following inclusion criteria: 1) Types of studies: prospective or retrospective studies reporting the HAS-BLED and ORBIT scores for predicting the bleeding risk; 2) Participants: non-valvular AF patients with VKA and non-VKA anticoagulants; and 3) Outcomes: major bleeding was defined based on the 2005 International Society on Thrombosis and Haemostasis [ISTH] criteria [8]. (i) fatal bleeding and/or (ii) symptomatic bleeding in a critical area or organ (intracranial, intraspinal, intraocular, retroperitoneal, intra-articular or pericardial, or intramuscular with compartment syndrome), and/or (iii) bleeding causing a decrease in the hemoglobin level of 20 g L^−1^ or more or leading to the transfusion of two or more units of whole blood or red cells.

Studies with insufficient data, not published in English, certain publication types (e.g., conference abstracts, letters, comments, case reports, and reviews) were excluded from this meta-analysis. For those duplicated studies, studies with the longest follow-up or largest sample size were included.

### Literature search

We systematically searched the Cochrane Library, Elsevier and PubMed electronic databases for studies reporting the HAS-BLED and ORBIT scores for predicting the bleeding risk. The included studies were published from January 2010 to June 2017 because the HAS-BLED score was first proposed in 2010 [7] and the ORBIT score was developed in 2015 [8]. Search terms included ‘atrial fibrillation’, ‘ORBIT’, ‘HAS-BLED’, ‘anticoagulation’ and ‘major bleeding’. We did not find other studies in the manual search.

### Data extraction and quality assessment of individual studies

Included studies were selected by two reviewers based on the search terms. The studies were prescreened by C.W., who read the titles and abstracts. The second round of selection involved the complete and careful review of articles by Y.Y., to confirm whether those studies described the bleeding events at each point or each stratification of the HAS-BLED and ORBIT scores. Discrepancies were resolved through discussion or consultation with a third reviewer (K.H.).

The Newcastle–Ottawa scale [17] was used to evaluate the quality of all included studies. It contains three parts: cohort selection, cohort comparability, and outcome assessment. Two authors independently assessed both the risk of bias and the quality of the seven studies in our meta-analysis.

### Statistical analysis

We used Review Manager Version 5.3 (Copenhagen, The Nordic Cochrane Centre, The Cochrane Collaboration, 2014) to analyze the data. Receiver operating characteristic curve (ROC) analysis, expressed by the C-statistic [95% confidence intervals (CIs)] of continuous variables, was applied to evaluate the diagnostic performance in the HAS-BLED and ORBIT scores [18–21]. The Z-statistic ($\frac{A_{Z1}-{Α}_{\mathrm{Ζ2}}}{\sqrt{SE_{1}^{2}+SE_{2}^{2}}}$) was used to assess the difference

between these two scores. *I*^2^ values ≤ 25%, 25% to ≤ 50%, and > 50% represented 'low', 'intermediate' and 'high' heterogeneity, respectively. When I^2^ values > 50%, a random-effects model was selected [22]. Both the HAS-BLED and ORBIT scores were divided into three risk stratifications. HAS-BLED = 0–1 and ORBIT = 0–2 were considered as low risk; HAS-BLED = 2 and ORBIT = 3 were defined as intermediate risk; and HAS-BLED ≥ 3 and ORBIT ≥ 4 were regarded as high risk (Table 1). Odds ratios (ORs) with their 95% CIs were chosen to compare the predictive value between these two scores. In addition, calibration analysis was used to assess the predictive accuracy of risk scores. The first published study reporting the ORBIT was a predictive model providing the adjusted bleeding rate (events/100 patient-years) across three risk classifications (low, 2.4%; intermediate, 4.7%; and high, 8.1%). Then, we calculated the predicted number of major bleeding events in the subsequent validated studies. The observed number of major bleeding events was collected from all the included studies. OR > 1 represented over-prediction, while OR < 1 represented under-prediction.

## RESULTS

### Description of the included studies and patients’ characteristics

**Figure 1:**
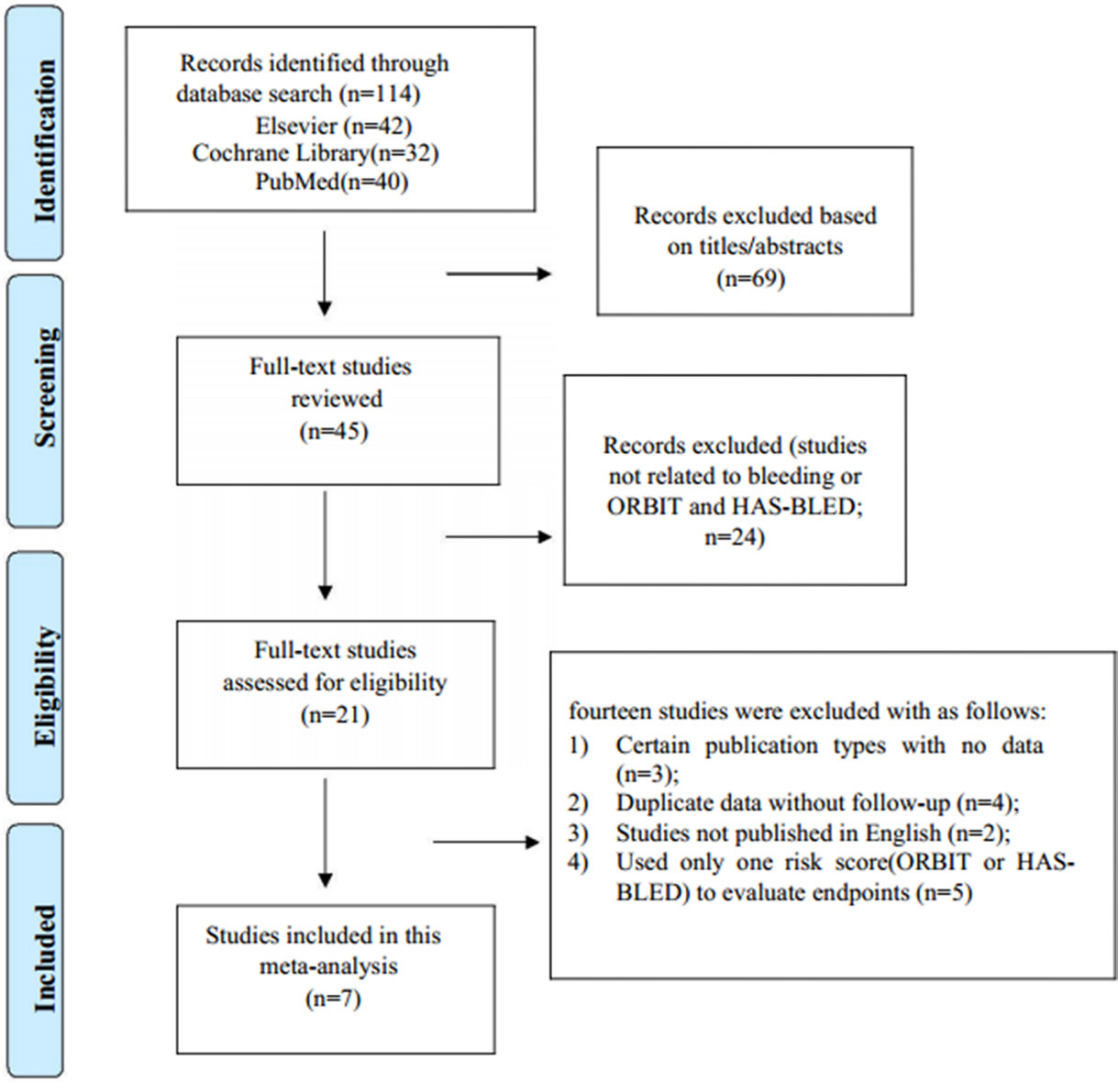
Study search diagram

A total of 114 studies were initially identified by the above-mentioned search strategies (42 in Elsevier, 32 in Cochrane Library, and 40 in PubMed). After reading the titles and abstracts, we excluded 69 studies. Next, 24 studies were removed because the studies did not relate to the bleeding events or the selected risk scores (ORBIT or HAS-BLED). Finally, 7 identified studies met our criteria [8, 10–15], and the others were excluded as follows: (i) Certain publication types with no data (*n* = 3); (ii) Duplicate data without follow-up (*n* = 4); (iii) Studies not published in English (*n* = 2); and (iv) Only one risk score (ORBIT or HAS-BLED) used to evaluate bleeding risk (*n* = 5) (Figure 1).

The participants of our seven included studies come from American, British, Spanish and Netherlands. [8, 10–15] (Table 2). We selected 8079 non-valvular AF patients with anticoagulant therapy (e.g., warfarin, apixaban, dabigatran and rivaroxaban) for further analyses and 348 of them had suffered from major bleeding finally. The 2005 ISTH criteria was used to define the major bleeding in our included studies. Furthermore, the quality of all included studies were evaluated by the Newcastle–Ottawa Scale. Eight or nine stars represented the high quality of these studies (Supplementary Table 1).

**Table 2:** Patients’ characteristics of the 7 included studies

Study	Data source	Follow-up	Patient number	Age (years)	Major Bleeding Definitions	Major Bleeding events	Anticoagulants
O’Brien E C [8]	USA (2010–2012)	Mean, 24 months	7411	75	2005 ISTH criteria	1353	Warfarin DOACs
Proietti M [10]	UK (–)	Median, 19 months	3551	72	NA	127	Warfarin
Senoo K [11]	Netherlands (2003–2005)	NA	2293	71	2005 ISTH criteria	NA	Warfarin
Esteve-Pastor M A [12]	Spain (2013–2014)	Mean, 12 months	1276	74	2005 ISTH criteria	46	Warfarin DOACs
Senoo K [13]	Netherlands (2003–2005)	Median, 10 months	2283	71	2005 ISTH criteria	74	Idraparinux
Abumuaileq R R [14]	Spain (2011–2013)	Mean, 11 months	911	75	2005 ISTH criteria	30	Warfarin
Caro M C [15]	Spain (2013–2014)	Mean, 22 months	969	76	2005 ISTH criteria	101	DOACs

Abbreviations: NA = not available; ISTH = International Society on Trombosis and Haemostasis.

DOACs = the new direct oral anticoagulants, apixaban, dabigatran and rivaroxaban.

### Discrimination analysis between the ORBIT and HAS-BLED scores

The first original article derived and validated the ORBIT score in the ORBIT-AF registry cohort and ROCKET-AF clinical trial populations [8]. The study by Proietti et al. did not provide the C-statistic [10]. Thus, there were a total of seven C-statistics of continuous variables for major bleeding in our 7 selected studies. Four showed that the ORBIT score had a higher C-statistic than the HAS-BLED [8, 12, 14]. The C-statistics ranged from 0.58 to 0.74 (median 0.62) for the ORBIT and from 0.59 to 0.68 (median 0.63) for the HAS-BLED, indicating that both scores had a modest predictive value for bleeding risk.

In our pooled analysis, the C-statistic was 0.65 (0.60, 0.69) for the ORBIT score and 0.63 (0.60, 0.66) for the HAS-BLED score. The Z-statistic between ORBIT and HAS-BLED was 0.72 (*p > 0.05*), showing that these two scores had a similar discriminative performance for bleeding risk (Supplementary Table 2).

### Major bleeding risks comparing ORBIT and HAS-BLED

#### Low risk category

**Figure 2:**
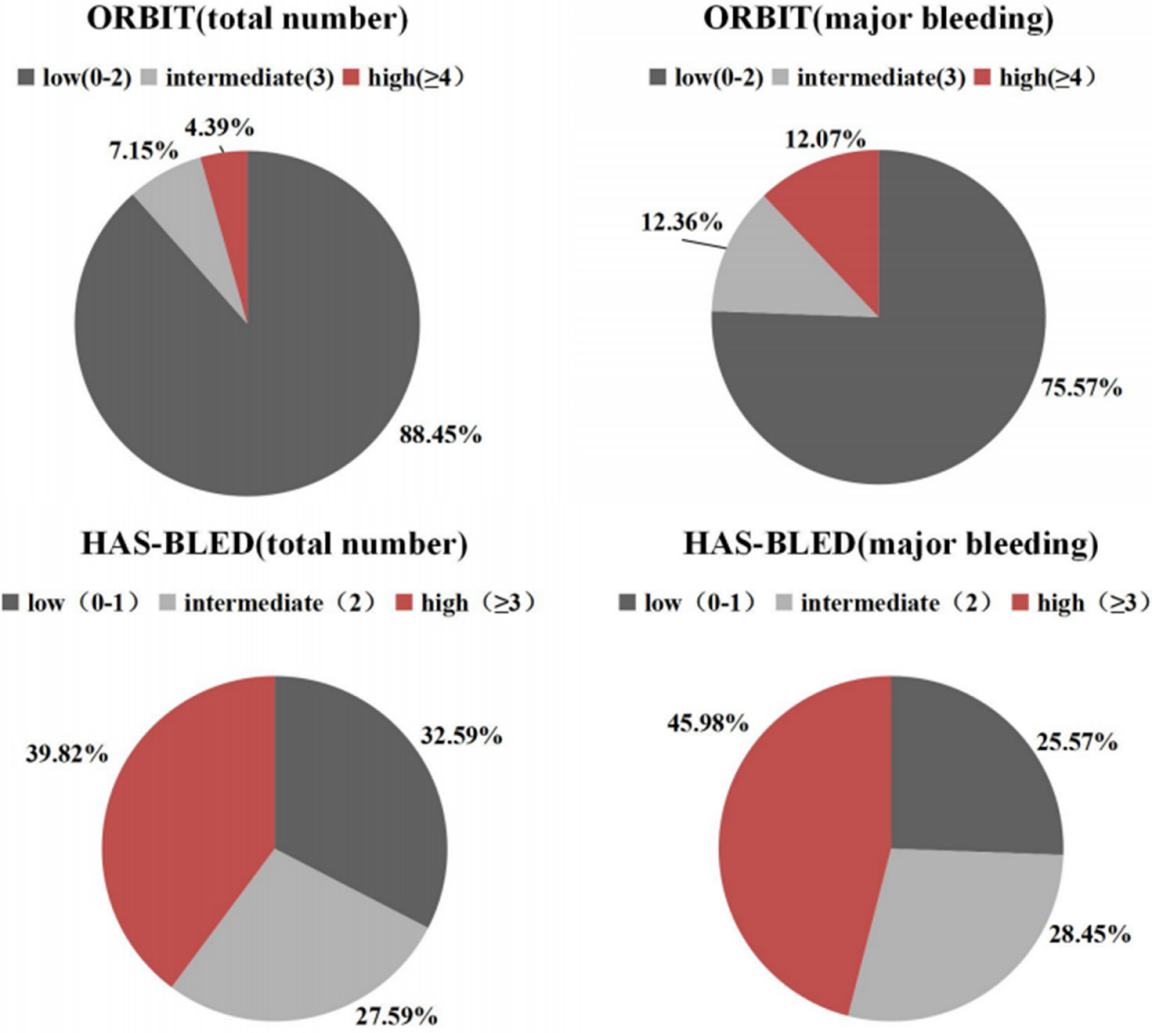
Population distribution in three strata for the ORBIT and HAS-BLED scores

The ORBIT score categorized 88.45% (7146/8079) of anticoagulated AF patients and 75.57% (263/348) of major bleeding events into the low risk category. With HAS-BLED, 32.59% (2633/8079) of total patients and 25.57% (89/348) of major bleeding were categorized as low-risk (Figure 2).

**Figure 3:**
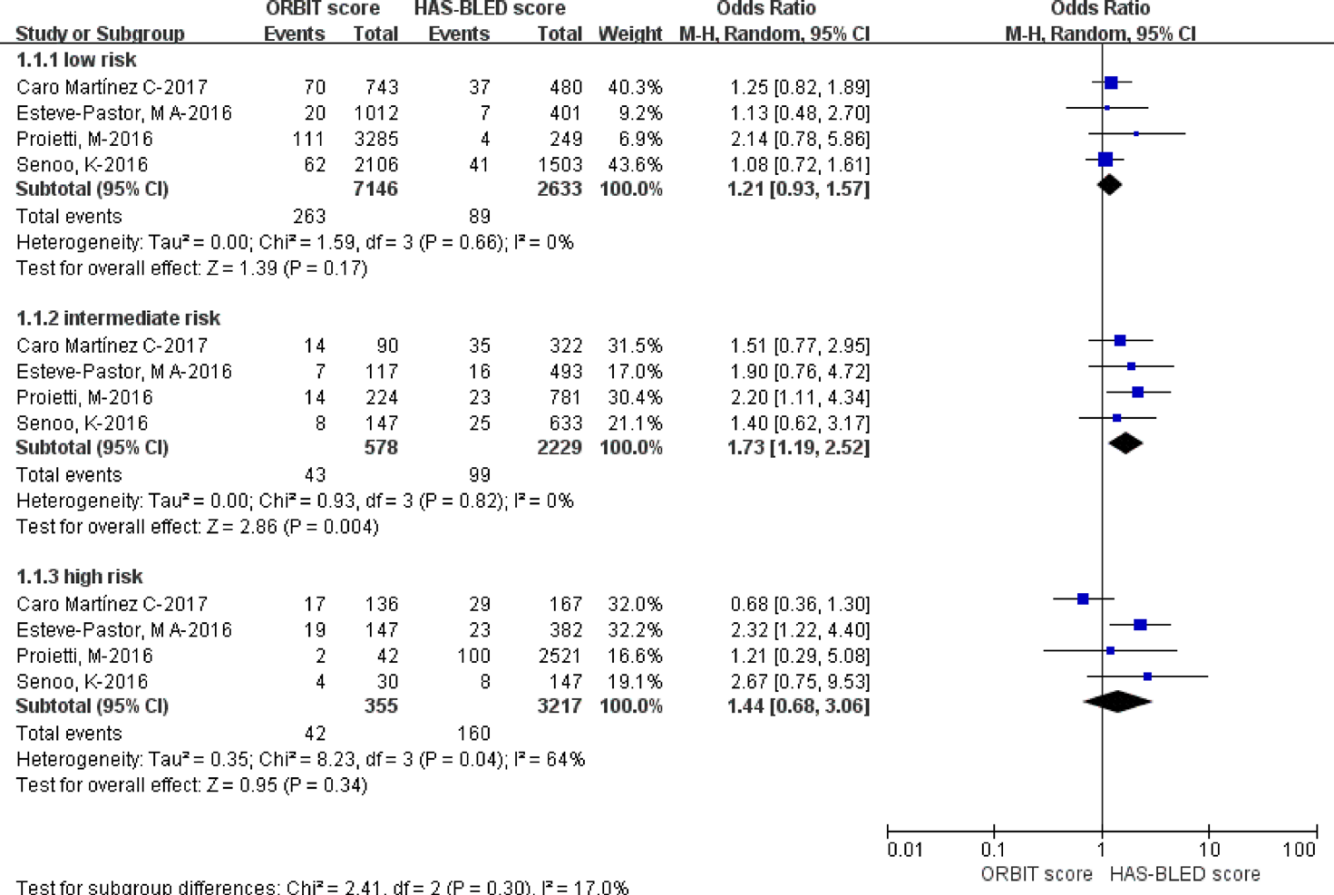
Comparison of major bleeding events in the ‘low’, ‘intermediate’ and ‘high’ risk categories based on the ORBIT and HAS-BLED scores Abbreviations: CI, confidence interval; df, degrees of freedom; ORBIT, older age [≥ 74 years], reduced hemoglobin/hematocrit/history of anemia, bleeding history, insufficient kidney function, and treatment with antiplatelet agents; HAS-BLED, hypertension, abnormal renal/liver function, stroke, bleeding history or predisposition, labile international normalized ratio [INR], elderly [age ≥ 65 years], and drugs/alcohol concomitantly; M-H, Mantel-Haenszel.

Our pooled analysis showed that the low-risk patients of ORBIT had a 1.21-fold greater risk of major bleeding events compared to those of HAS-BLED (OR = 1.21; 95% CI: 0.93–1.57; *p = 0.17*; *I*^*2*^ = 0%; Figure 3).

#### Intermediate risk category

In this strata, 7.15% (578/8079) of anticoagulated AF patients and 12.36% (43/348) of major bleeding events were categorized as intermediate risk when stratified by the ORBIT score. By contrast, the HAS-BLED score categorized 27.59% (2229/8079) of anticoagulated AF patients and 28.45% (99/348) of major bleeding events as intermediate-risk (Figure 2).

In our pooled analysis, the intermediate-risk patients of ORBIT had a 1.73-fold increased risk of major bleeding events when compared with those of HAS-BLED (OR = 1.73; 95% CI: 1.19–2.52; *p = 0.004*; *I*^*2*^ = 0%; Figure 3).

#### High risk category

The ORBIT score only categorized 4.39% (355/8079) of anticoagulated AF patients and 12.07% (42/348) of major bleeding events as high-risk. With HAS-BLED, 39.82% (3217/8079) of patients and 45.98% (160/348) of major bleeding events were categorized as high-risk (Figure 2).

Our pooled analysis showed that the high-risk patients of ORBIT had a 1.44-fold elevated risk of major bleeding events compared to HAS-BLED (OR = 1.44; 95% CI: 0.68–3.06; *p = 0.34*; *I*^*2*^ = 64%; Figure 3).

### Calibration analysis of the ORBIT score

**Figure 4:**
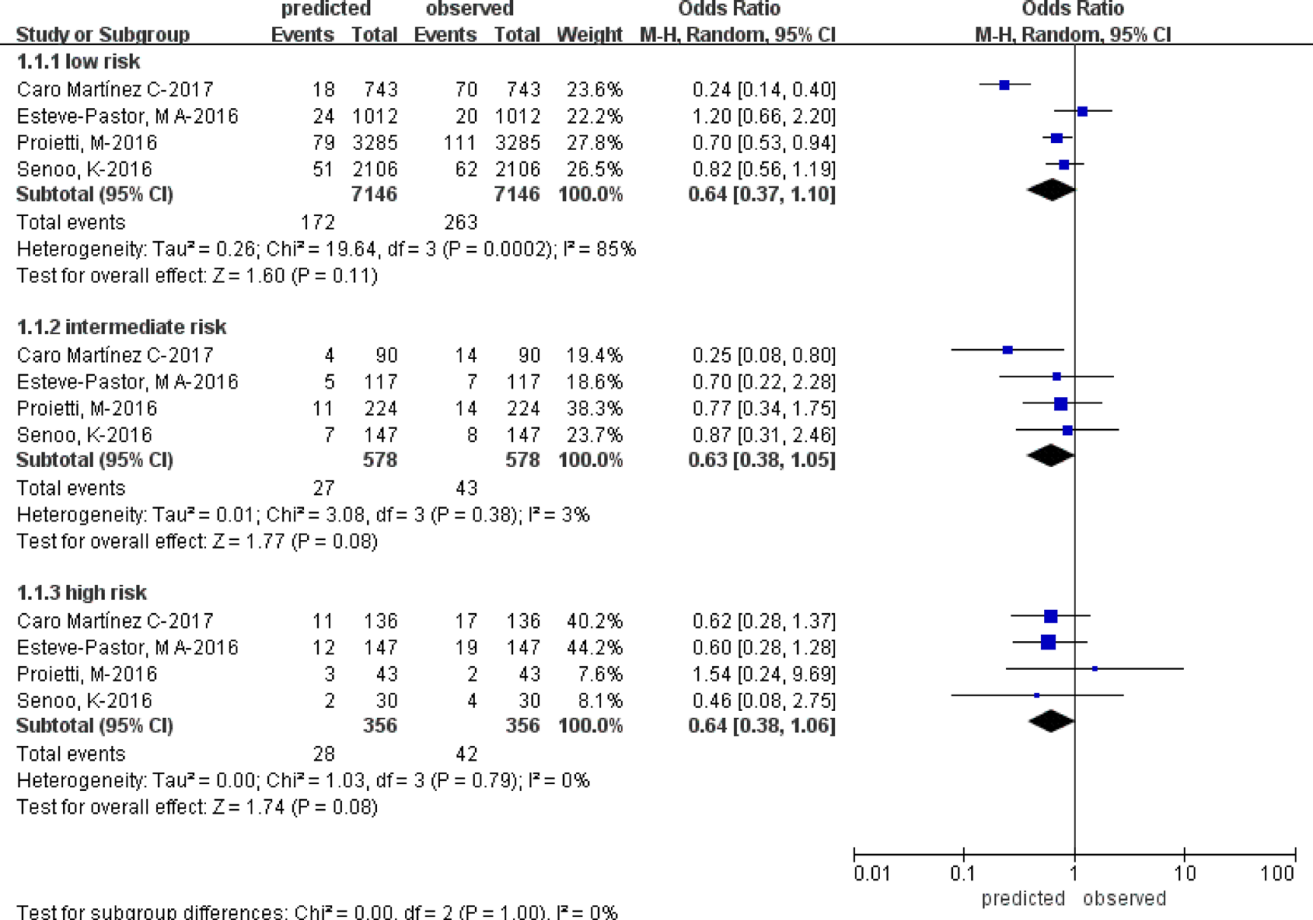
Forest plot showing calibration analysis of the ORBIT score

Four studies [10, 12, 13, 15] were included in the calibration analysis. All the OR values were less than 1.0 across the three risk strata [low (OR = 0.64; 95% CI: 0.37–1.10; *p = 0.11; I*^*2*^ = 85%); intermediate (OR = 0.63; 95% CI: 0.38–1.05; *p = 0.08; I*^*2*^ = 3%); and high (OR = 0.64; 95% CI: 0.38–1.06; *p = 0.08; I*^*2*^ = 0%)] (Figure 4).

The ORBIT score under-predicted risk in low, intermediate and high strata, although there were no differences between the observed and predicted events in the three risk stratifications.

## DISCUSSION

Based on our pooled C-statistic of continuous variables, both the ORBIT and HAS-BLED scores had a modest discriminatory capacity for predicting major bleeding risks. Although our pooled analysis showed that the ORBIT score had an elevated risk of major bleeding in all the three risk strata when compared with HAS-BLED, the *P* > 0.05 represented no significant differences in low and high stratifications. Compared with the HAS-BLED, more anticoagulated AF patients and major bleeding events were categorized as low risk when using ORBIT. Moreover, the ORBIT score under-predicted risk in all the three strata, but the *p* value > 0.05 represented that the ORBIT score was an adequate model calibration in predicting bleeding events.

### Adding time in therapeutic range to the ORBIT scores

In recent studies of Vitamin K antagonist (VKA, e.g. ,warfarin) cohorts, the ORBIT score also underperformed in bleeding prediction compared to HAS-BLED, by not considering the ‘labile INR criterion’ [10, 11]. This is relevant as VKAs are still widely used as the preferred OAC worldwide. Indeed, the ‘labile INR criterion’, as measured by time in therapeutic ranges (TTR) [23, 24], is one of the most powerful criteria for bleeding prediction, and is incorporated within the HAS-BLED score. Abumuaileq et al.found that the ORBIT score with TTR value (C-statistic = 0.74; 95% CI:0.70–0.76) had a higher C-statistic values than the original ORBIT (C-statistic = 0.66; 95% CI, 0.63–0.69) for major bleeding [14]. In the study by Senoo et al., the use of the ORBIT score with TTR values compared with the original ORBIT significantly reclassified +34.8% of the population for major bleeding and +26.0% for any clinically relevant bleeding [11]. According to the study of Proietti et al., when compared with the original ORBIT score, the ORBIT score with TTR values significantly reclassified +25.08% of the population for major bleeding [10]. Thus, the predictive ability of the ORBIT score with TTR values was improved compared to that of the original ORBIT score.

### Net reclassification improvement (NRI) analysis of the ORBIT score

Nonetheless, a C-statistic cannot correctly and adequately assess the diagnostic ability, and other parameters must be added [25]. The net reclassification improvement (NRI) is a metric that helps us to understand the improvement between event and non-event categories. When a choice of care is presented above or below the given cut-off, the NRI value is better than the C-statistic when evaluating the predictive ability [25, 26]. Three included studies in our meta-analysis used the NRI. For example, Abumuaileq et al. [14] and Proietti et al. [10] found that the ORBIT score reclassified −1.85% and −0.77% of the population for major bleeding (*p* = 0.9 and 0.392, respectively) when compared with the HAS-BLED. Senoo et al. [13] reported a positive reclassification for any clinically relevant bleeding (NRI: +15.6%, *p = 0.007*) with the HAS-BLED score when compared with the ORBIT. A significant positive NRI value indicated that the HAS-BLED score performed better than the ORBIT in predicting bleeding events. It was not possible to complete a pooled analysis because of the limited NRI data.

### ORBIT versus HAS-BLED score

Since the HAS-BLED score was first described in 2010, it has been validated in various populations, including AF and non-AF cohorts and various drug treatments (e.g., aspirin, VKA [27] and non-VKA anticoagulants [28–30]) and no antithrombotic therapy cohorts. The HAS-BLED score performs better than other scores in predicting major bleeding and ICH in an Asian/Chinese AF population [31]. For the ORBIT score, O’Brien and colleagues chose the five strongest predictors from a full continuous ORBIT bleeding model, which included numerous independent predictors of major bleeding [8]. In our pooled analysis, we found that this new score categorized more anticoagulated AF patients and major bleeding events as low risk when compared with the HAS-BLED score. And in clinical practice, the physician always pay more attention to the ‘high risk’ anticoagulated AF patients, they might timely adjust the dose of anticoagulant or even discontinue anticoagulant for the patients. In contrast, the ‘low risk’ anticoagulated AF patients might get less attention. Unfortunately, the ORBIT score did not meet expectations for evaluating bleeding risks, and several studies have found that the ORBIT only shows a modest or even worse predictive ability when compared with other existing scores [10–15].

#### Limitations

Several evident limitations are in our meta-analysis. First, heterogeneity always exists in three main forms: clinical, methodological and statistical [32], and the inconsistency might have derived from the limited amount of data, variety in study designs and the various types of anticoagulants used (e.g., warfarin, dabigatran, rivaroxaban, and apixaban ). Second, the types of included studies were prospective or retrospective studies, and large prospective studies are necessary to validate our findings.

## CONCLUSIONS

In conclusion, our systematic review and meta-analysis showed that the ORBIT score does not superior than the HAS-BLED in predicting major bleeding events of anticoagulated AF patients. Furthermore, when compared with the HAS-BLED score, this new bleeding risk score categorized more anticoagulated AF patients and major bleeding events as low risk.

## SUPPLEMENTARY MATERIALS TABLES


