# Supplementary material for: Comparing the ORBIT and HAS-BLED bleeding risk scores in anticoagulated atrial fibrillation patients: a systematic review and meta-analysis
Source: Oncotarget. 2017 Aug 3;8(65):109703–11. doi: 10.18632/oncotarget.19858 (PMC5752553; doi:10.18632/oncotarget.19858)
Supplement: Supplementary file 1 [file oncotarget-08-109703-s001.pdf]

Comparing the ORBIT and HAS-BLED bleeding risk scores in anticoagulated atrial fibrillation patients: a systematic review and meta-analysis

SUPPLEMENTARY MATERIALS

Supplementary Table 1: Quality assessment of all included studies

	Selection	Comparability	Outcome
O'Brien E C-2016 [1]	****	**	***
Proietti M-2016 [2]	****	**	**
Senoo K-2016 [3]	****	**	**
Esteve-Pastor M A-2016 [4]	****	**	***
Senoo K-2016 [5]	****	**	***
Abumuaileq R R-2016 [6]	****	**	***
Caro M C-2017 [7]	****	**	***

Supplementary Table 2: C-statistic of all included studies

Bleeding risk score	Study-ID	C-Statistic	Synthesis of C-Statistic (95% CI)
ORBIT	O'Brien E C (ORBIT-AF cohort)-2015 [1]	0.67 (0.64, 0.69)	0.65 (0.60, 0.69)
	O'Brien E C (ROCKET-AF cohort)-2015 [1]	0.62 (0.6, 0.64)	
	Senoo K-2016 [3]	0.61 (0.51, 0.7)	
	Esteve-Pastor M A-2016 [4]	0.7 (0.62, 0.77)	
	Senoo K-2016 [5]	0.58 (0.52, 0.65)	
	Abumuaileq R R-2016 [6]	0.74 (0.71,0.77)	
	Caro M C-2017 [7]	0.59 (0.56, 0.62)	
HAS-BLED	O'Brien E C (ORBIT-AF cohort)-2015 [1]	0.64 (0.62, 0.67)	0.63 (0.60, 0.66)
	O'Brien E C (ROCKET-AF cohort)-2015 [1]	0.59 (0.57, 0.61)	
	Senoo K-2016 [3]	0.65 (0.56, 0.73)	
	Esteve-Pastor M A-2016 [4]	0.63 (0.56, 0.71)	
	Senoo K-2016 [5]	0.59 (0.53, 0.65)	
	Abumuaileq R R-2016 [6]	0.68 (0.64,0.71)	
	Caro M C-2017 [7]	0.62 (0.59, 0.65)	

REFERENCES

1. O'Brien EC, Simon DN, Thomas LE, Hylek EM, Gersh BJ, Ansell JE, Kowey PR, Mahaffey KW, Chang P, Fonarow GC, Pencina MJ, Piccini JP, Peterson ED. The ORBIT bleeding score: a simple bedside score to assess bleeding risk in atrial fibrillation. Eur Heart J. 2015; 36:3258–64.
2. Proietti M, Senoo K, Lane DA, Lip GY. Major Bleeding in Patients with Non-Valvular Atrial Fibrillation: Impact of Time in Therapeutic Range on Contemporary Bleeding Risk Scores. Sci Rep. 2016; 6:24376. https://doi.org/10.1038/srep24376.
3. Senoo K, Proietti M, Lane DA, Lip GY. Evaluation of the HAS-BLED, ATRIA, and ORBIT Bleeding Risk Scores in Patients with Atrial Fibrillation Taking Warfarin. Am J Med. 2016; 129:600–07. https://doi.org/10.1016/j.amjmed.2015.10.001.
4. Esteve-Pastor MA, García-Fernández A, Macías M, Sogorb F, Valdés M, Roldán V, Muñiz J, Badimon L, Roldán I, Bertomeu-Martínez V, Cequier Á, Lip GY, Anguita M, Marín F, and FANTASIIA Investigators. Is the ORBIT Bleeding Risk Score Superior to the HAS-BLED Score in Anticoagulated Atrial Fibrillation Patients? Circ J. 2016; 80:2102–08. https://doi.org/10.1253/circj.CJ-16-0471.
5. Senoo K, Lip GY. Predictive abilities of the HAS-BLED and ORBIT bleeding risk scores in non-warfarin anticoagulated atrial fibrillation patients: An ancillary analysis from the AMADEUS trial. Int J Cardiol. 2016; 221:379–82. https://doi.org/10.1016/j.ijcard.2016.07.100.
6. Abumuaileq RR, Abu-Assi E, Raposeiras-Roubin S, Rodríguez-Mañero M, Peña-Gil C, González-Juanatey JR. Comparison Between 3 Bleeding Scoring Systems in Nonvalvular Atrial Fibrillation Patients. What Can the New ORBIT Score Provide? Rev Esp Cardiol (Engl Ed). 2016; 69:1112–14. https://doi.org/10.1016/j.rec.2016.05.022.
7. Caro MC, Andreu CJ, Flores BP, Valdes M, Bailen LJ, Manzano FS. Comparison of Bleeding Risk Scores in Patients With Nonvalvular Atrial Fibrillation Starting Direct Oral Anticoagulants. [Article in English, Spanish]. Rev Esp Cardiol (Engl Ed). 2017; 70:878–880. https://doi.org/10.1016/j.rec.2017.01.021.
